# Supplementary material for: Data management and sharing: Practices and perceptions of psychology researchers
Source: PLoS One. 2021 May 21;16(5):e0252047. doi: 10.1371/journal.pone.0252047 (PMC8139478; doi:10.1371/journal.pone.0252047)
Supplement: Attachment — Submitted filename: Response to reviewers.docx [file pone.0252047.s002.docx]

April 2021

We respectfully submit a revised version of our manuscript that has been revised based on the requested revisions and suggestions of your editorial staff and the peer reviewers. Please see below for our point by point responses.

Thank you.

**"The submitted version of this paper has been uploaded to PsyArXiv.
Borghi, J.A. & Van Gulick, A.E. (2020). Data Management and Sharing: Practices and Perceptions of Psychology Researchers. PsyArXiv,** [**https://doi.org/10.31234/osf.io/7g3ae**](https://doi.org/10.31234/osf.io/7g3ae)**"**

**Please clarify whether this publication was peer-reviewed and formally published. If this work was previously peer-reviewed and published, in the cover letter please provide the reason that this work does not constitute dual publication and should be included in the current manuscript.**

We have clarified in our cover letter that the version of this paper on PsyArXiv is a preprint and was neither peer reviewed or formally published.

**5. Please include captions for your Supporting Information files at the end of your manuscript, and update any in-text citations to match accordingly. Please see our Supporting Information guidelines for more information:** [**http://journals.plos.org/plosone/s/supporting-information**](http://journals.plos.org/plosone/s/supporting-information)**.**

We have added revised the name of our supplemental file on line 156 and added a caption starting on line 432.

**I was impressed that 8,474 unique researchers had been invited to participate in the survey, but I was disappointed to see that only 274 completed the survey, which represents merely a ~3% response rate. Could the authors speculate on why their response rate was so low? Does MailChimp provide any data on how many invitation emails were actually received and/or opened? Did many respondents start but not complete the survey?**

Thank you for this question, we have adjusted the language in lines 175 to 181 clarifying that we believe our strategy of sending unprompted e-mails is responsible for the low response rate. We do not have data on how many e-mails were actually received, but we believe this number is still relatively high given that we used e-mails scraped from articles published 1 or 2 years prior to the distribution of our survey. In our discussion section (line 373) we note how our survey – which was explicitly advertised as being about data management in psychology – may have disproportionately attracted psychology researchers with better than average data management practices.

**The areas where inferential statistics were performed come across as being somewhat “cherry-picked”. Could the authors provide better motivation for these statistical analyses?**

We have added a sentence (line 227) clarifying that these analyses were in line with those conducted in our earlier survey.

**Line 174, “After filtering”: I’m not sure what this means. Does this mean “After deduplication”?**

This refers to the number of participants who met our inclusion criteria. We have revised this sentence to clarify this point.

**Figure 1: I recommend providing verbal labels in the graph legends rather than numerical labels (e.g., 1, 2, 3, . . . ) to facilitate reader comprehension.**

Thank you for this comment, we did not provide verbal labels for every value in survey text, so we did not feel it appropriate to add them here. However, we have adjusted the figure legends to further facilitate reader comprehension.

**Minor correction needed in line 296: Figure 4 should be Table 4.**

Thank you. We have corrected this error, now on line 306.

**As suggestion: it would be good to visualize the information in tables 2, 3, 4 as graphs.**

Thank you for this suggestion, we decided to use tables to communicate this information rather than graphs because we felt that a graph would not add any additional information.
